# Direction Specific Biases in Human Visual and Vestibular Heading Perception

**DOI:** 10.1371/journal.pone.0051383

**Published:** 2012-12-07

**Authors:** Benjamin T. Crane

**Affiliations:** 1 Department of Otolaryngology, University of Rochester, Rochester, New York, United States of America; 2 Department of Neurobiology and Anatomy, University of Rochester, Rochester, New York, United States of America; 3 Department of Bioengineering, University of Rochester, Rochester, New York, United States of America; VU University Amsterdam, The Netherlands

## Abstract

Heading direction is determined from visual and vestibular cues. Both sensory modalities have been shown to have better direction discrimination for headings near straight ahead. Previous studies of visual heading estimation have not used the full range of stimuli, and vestibular heading estimation has not previously been reported. The current experiments measure human heading estimation in the horizontal plane to vestibular, visual, and spoken stimuli. The vestibular and visual tasks involved 16 cm of platform or visual motion. The spoken stimulus was a voice command speaking a heading angle. All conditions demonstrated direction dependent biases in perceived headings such that biases increased with headings further from the fore-aft axis. The bias was larger with the visual stimulus when compared with the vestibular stimulus in all 10 subjects. For the visual and vestibular tasks precision was best for headings near fore-aft. The spoken headings had the least bias, and the variation in precision was less dependent on direction. In a separate experiment when headings were limited to ±45°, the biases were much less, demonstrating the range of headings influences perception. There was a strong and highly significant correlation between the bias curves for visual and spoken stimuli in every subject. The correlation between visual-vestibular and vestibular-spoken biases were weaker but remained significant. The observed biases in both visual and vestibular heading perception qualitatively resembled predictions of a recent population vector decoder model (Gu et al., 2010) based on the known distribution of neuronal sensitivities.

## Introduction

Visual heading perception is influenced by optic flow [1]–[4]. It has classically been studied in one of two ways: either as discrimination in which a heading is compared with a reference position using a forced choice task (i.e. is the stimulus right or left relative to straight ahead or a reference stimulus) [5]–[9] or estimation in which the subject directly reports the perceived heading using a pointing device such as a cursor [10], [11] or physical pointer [12]. Both of these methods have focused on headings near straight ahead.

In prior visual heading estimation tasks which studied pure translation the perception was usually within a few degrees of the actual heading with some studies reporting underestimation (the perceived heading is closer to the fore-aft axis than actual) which was usually small [10], [11], [13], [14], and others reporting slight [15] or large [12] overestimation in which the perceived heading is further from the fore-aft axis than actual. For these estimation tasks, the horizontal range tested was limited: in one instance to as large as 90° [15] but usually much less at ±25° [10], ±20° [12] ±15° [15] or less [11], [14]. To the knowledge of the author, a visual heading estimation task has not previously been published using a full range of headings in the horizontal plane.

The range of headings included has important implications for perception. When, the visual focus of expansion (FOE) is within the field of view (FOV), its location gives the heading direction [9]. When the FOE is outside the FOV heading can be determined using triangulation of vectors determined from motion of fiducial points and is potentially less accurate [16] although experiments which have examined this question have found the accuracy to be similar when the FOE is in or outside the FOV [11], [15]. Although theoretically the size of the FOV influences heading accuracy [16], other factors may be predominant as headings estimated from a 112°FOV and similar accuracy to estimates made through a 5 or 10° aperture [11].

Another potentially more important issue is the range of heading stimuli tested and the range of responses permitted. Prior to viewing each stimulus subjects likely had an internal model of the range of possible stimuli and inferred that stimuli in this range would be most likely. Even if this range were not explicitly given to the subject, it might be inferred from the range of stimuli experienced in earlier trials or the range of responses permitted. In previous studies of visual heading estimation both the range of responses and stimuli were limited [10]–[12]. Bayesian theory provides a quantitative basis for this prior estimate of stimulus distribution that influenced their subsequent perception [17]–[19]. Thus if subjects expects headings within a fixed range, they would be unlikely to perceive headings outside this range [20]. The current study avoids the issue of having the stimuli limited to a range of angles by presenting stimuli which are uniformly distributed about a full 360° in the horizontal plane such that the FOE is not present on the screen for 74% of stimuli. Responses are similarly not limited. With this protocol, subjects are less likely to limit their responses based on a prior expectation that the range of stimuli is limited.

In most real world experiences visual and vestibular heading cues are available. Study of vestibular heading has been more limited than study of visual heading. Most of these have studied discrimination relative to a reference position in humans [8], [21] and non-human primates [8], [22], [23]. Unlike estimation methods, discrimination does not reveal internal biases since two stimuli compared. There have also been studies of human heading estimation in darkness [24] and with and without visual cues [12]. These previous studies of vestibular heading perception in darkness suggested perceived headings are slightly underestimated relative to fore-aft. However, like the studies on visual heading estimation, these vestibular studies limited the potential stimuli to a small number of potential headings in a narrow horizontal range.

The prior work on heading estimation does not take in to account any potential biases in spatial cognition, haptic, or motor influences that may be independent of sensory stimulation. It is possible that some of the bias in heading perception may be due to an internal representation of space that is itself biased. Also, the method used to report the perceived heading will have a haptic and motor component. In the current study, efforts to control for this are made by having subjects orient a pointer towards a verbally spoken angular heading.

Visual and vestibular heading estimation may not be independent as both are represented in medial superior temporal area (MSTd) of the cortex [25]–[27] which is likely a key area in determining heading perception [4], [8], [28], [29]. Recent modeling of this area using a population vector decoder (PVD) as well as a maximum likelihood (ML) estimate model been used to explain the increased precision in determining headings near straight ahead [8]. The PVD model is relatively simple in that each neuron essentially weighs in on its preferred direction based on the magnitude of its response [30]. Such a PVD model has previously been used to explain visual pursuit based on MSTd activity [31]. However the PVD model has limitations in that estimates may be biased toward directions with more neurons responding in that preferred direction [32]. To correct for this the ML model applies probability theory to a population of neurons to find the maximum likelihood for a set of parameters [32]. Although the ML method is useful in analysis of experimental data it is not intended as a biologically plausible neuronal computation algorithm [32]. The PVD model is relatively simple and thus more biologically plausible. In comparing the PVD and ML models to heading estimation in MSTd, the PVD predicted that both visual and vestibular heading would be overestimated by large amounts at eccentric headings, while the ML model predicted headings without a direction specific bias [8]. The data used by Gu et al. to develop these models tested heading discrimination but not heading estimation and thus could not differentiate between the predictions of these competing models. The current study looks for the possible biases predicted by the PVD model by measuring visual and vestibular heading estimates over the full range of horizontal. It was found that perceived heading relative to straight ahead is overestimated with both visual and vestibular stimuli similar to what the PVD model predicts.

## Materials and Methods

### Ethics Statement

The research was conducted according to the principles expressed in the Declaration of Helsinki. Written informed consent was obtained from all participants. The protocol and written consent form were approved by the University of Rochester Research Science Review Board (RSRB).

### Equipment

Motion stimuli were delivered using a 6-degree-of-freedom motion platform (Moog, East Aurora, NY, model 6DOF2000E) similar to that used in other laboratories for human motion perception studies [21], [33], [34] and previously described in the current laboratory [35]. Subjects were seated in a padded racing seat (Corbeau, Sandy UT, model FX-1) mounted on the platform. A four-point racing style harness held the body in place. The head was held in place using an American football helmet (Riddell, Eyria, OH) with the facemask removed to improve visibility. Helmets were available in 6 sizes to allow each subject to be fit appropriately. The helmet had an inflatable liner to insure a sung fit. Once the subject was seated the helmet was firmly pushed back against hard rubber pads and a strap was used hold the helmet against the pad and prevent decoupling. A second rigid point of attachment on the side of the helmet further prevented any decoupling. The head was held in position so that the body midline and external auditory canals were directly over the center of the platform.

During both visual and vestibular stimuli, an audible white noise was reproduced from two platform-mounted speakers on either side of the subject as previously described [35]. The intensity of the masking noise varied with time as a half-sine wave so that the peak masking noise occurred at the same time the peak velocity was reached. This created a masking noise similar to the noise made by the platform. Although no masking noise was needed for the visual condition it was still used for consistency. For clarity, masking noise was not used in trials when the heading was spoken.

Responses were collected using a two-button control box with a dial in the middle that could be freely rotated in the horizontal plane without any discontinuity points. The box with the dial was mounted 20 cm anterior to the subject just above waist level below the viewing screen. The dial was not visible during the experiment and orientation was by feel. The dial was connected to a 14 bit rotary encoder (Contelec, model VertX1332, Biel Switzerland) which was calibrated to a <0.1° angular resolution.

The two buttons at either end had the same function. After an audible tone indicated that the next stimulus was ready a button could be pressed to deliver the stimulus. After the stimulus was delivered a series of two tones indicated the perceived heading direction should be selected. After the heading was selected, one of the buttons was pressed again so the subject could signal that they had finished their selection. The dial remained in the position the subject left it for the next stimulus presentation. Although the heading direction and residual position from the previous trial may have influenced the response to the subsequent trial, any effect likely evened out in the aggregate data as the stimuli were given in a random order which was different for each block of trials. There were no explicit orientation markers on the dial (such as a divot at the zero position) although the dial was mounted in a rectangular box so the edges of the box might have served as reference positions.

### Stimulus

The visual and vestibular stimuli consisted of a 2s (0.5 Hz) sine wave in acceleration. The stimulus can be described in the acceleration (*a*(*t*)), velocity (*v*(*t*)), or position (*d*(*t*)) domains given the frequency in Hz (*F*) and total displacement (*D*) (equations 1–3). These motion profiles were chosen because they contain no discontinuities in acceleration, velocity, or position, and they have previously been used for threshold determination [33], [35], [36].
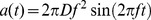
(1)

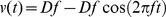
(2)

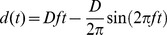
(3)


Visual stimuli were presented on a color LCD screen measuring 115.6 by 64.8 cm with a resolution of 1920×1080 pixels (Samsung model LN52B75OU1FXZA). The subject was seated 50 cm from the screen that filled a 98° horizontal field of view. A fixation point consisted of a 2×2 cm midline cross at eye level. The visual stimulus consisted of a star field which simulated movement of the observer through a random-dot cloud. Each star consisted of a triangle 0.5 cm in height and width at the plane of the screen, adjusted appropriately for distance. The star density was 0.01 per cubic cm. The depth of the field was 130 cm. Visual coherence was fixed at 100%. Disparity was provided using red-green anaglyph glasses made with Kodak (Rochester, NY) Wratten filters #29 (dark red) and #61 (deep green). The colors were adjusted such that the intensities of the two were similar when viewed through the respective filters and rejection ratio was better than ten fold.

Five stimulus types were tested: Motion in darkness with nothing visible and no fixation point (NF), this type of trial had a displacement of 16 cm with a peak velocity of 16 cm/s and peak acceleration of 25 cm/s/s. A similar trial was done with a small fixation point visible on a video screen (FP). A set of trials was done in complete darkness with the movement designed to be sub-threshold (ST) with a displacement of 1 cm, peak velocity of 1 cm/s, and acceleration of 1.6 cm/s/s. A visual (V) stimulus displayed the pattern of motion expect for this movement through a star field. The final test type was a spoken (S) stimulus in which a computer generated voice would speak the desired heading relative to straight ahead (i.e., “45 degrees right”, or “135 degrees left.”) which was done in darkness with no platform motion. Each block of trials consisted of stimulus presentations of a single type, to keep trial blocks at a reasonable length and maintain alertness. The order of stimulus blocks was varied between subjects.

Each block consisted of 72 stimulus presentations: The stimuli were headings at 5° increments such that all 360° was equally represented. The headings were delivered in random order throughout the trial block with each heading delivered once. Test types NF, FP, V, and S were each repeated twice for each subject. After examining the data it was felt that consistent results were found after two repetitions and additional repetitions were not needed. Test ST was only done once. To maintain subject alertness, testing was broken up into at least 2 sessions on different days. Subjects were not required to complete a certain number of trial blocks in each session but 4–5 trial blocks were typical.

### Experimental Procedure

Subjects were instructed that each stimulus would move or simulate motion along a vector in the horizontal plane, or in case of the spoken (S) test a spoken heading would be heard. Prior to testing subjects were shown how to orient the dial. Occasionally, subjects were seen to make systematic errors early in a session such as identifying the direction the star field was moving rather than their direction through the star field in the visual system. These types of errors were rare and identified in the first few trials. When this occurred the subject was given further instruction and the trial block was restarted. Prior to the spoken condition subjects were given a brief orientation to the cardinal axes (i.e. 0° is forward, 90° is right, etc.) It was made clear to subjects that the spoken angles were relative to straight ahead, which was defined as 0°. In visual and vestibular conditions an audible beep marked the end of the stimulus alerting the subject to orient a dial towards the perceived direction. Subjects were encouraged to guess if uncertain. The experiment was practiced a few times in the light to ensure comprehension of the task prior to data collection in darkness. Two subjects (#3 and #9) were familiar with the design of the experiment, the other subjects had participated in previous experiments in the lab using the motion platform but were otherwise naïve to the design and purpose of the experiment.

Prior to stimulus delivery the subject heard a 500 Hz, 0.125s single tone to signal that the next stimulus was ready and the start button could be pressed. The stimulus was delivered immediately after the subject pressed the start button. After the stimulus was delivered, two 0.125 s tones were played in rapid succession to indicate the stimulus had been delivered and the perceived direction could be entered. If no response was entered a “timeout sound” was played (a low frequency buzz). The time out occurred at 3 s for motion stimuli and at 10 s for spoken stimuli, a task which tended to take subjects longer. After either a response or timeout, the platform returned to the center starting position using a motion profile similar to the stimulus but taking 2.5 s.

The experiment was repeated using stimuli that were limited to 5° increments in the range of ±45°. These experiments were done after the experiments that included a 360° range of headings, in a subset of six subjects (1, 2, 3, 7, 8, and 9). Conditions tested included visual (V), vestibular without no fixation (NF), and spoken. To make this control experiment as similar as possible to prior experiments in which the responses were limited by the screen size the responses were initially mechanically limited to a ±45° range, the size of the screen in a prior study [10]. Other than the mechanical limit on responses no explicit feedback was given. This was also felt to be most consistent with previous studies [10], [11] in which the range of headings which could be reported was also limited. The conditions were repeated without limits on the responses, but with only stimuli in the ±45° range tested. As with the other experiments the headings were delivered in random order. In V and NF each heading was presented three times, and in the spoken condition it was presented twice.

### Subjects

A total of 10 subjects (3 female, 1 left handed) participated in the experiment. Ages ranged from 21 to 66 (37±16, mean±standard deviation). All 9 blocks of trials using a 360° range of stimuli were usually completed in two sessions lasting no more than 90 minutes each with breaks between blocks of trials. In the subset of 5 subjects, in whom additional experiments used a ±45° range of stimuli, this testing was in a single session on a separate day. The order of the blocks was randomized within each session, except trials of type NF or FP were completed first. This was done so that if the subject did not understand the instructions it would be obvious early in the session.

Subjects were screened prior to participation for normal peripheral vestibular function and hearing as previously described [37].

### Analysis

**Figure 1:**
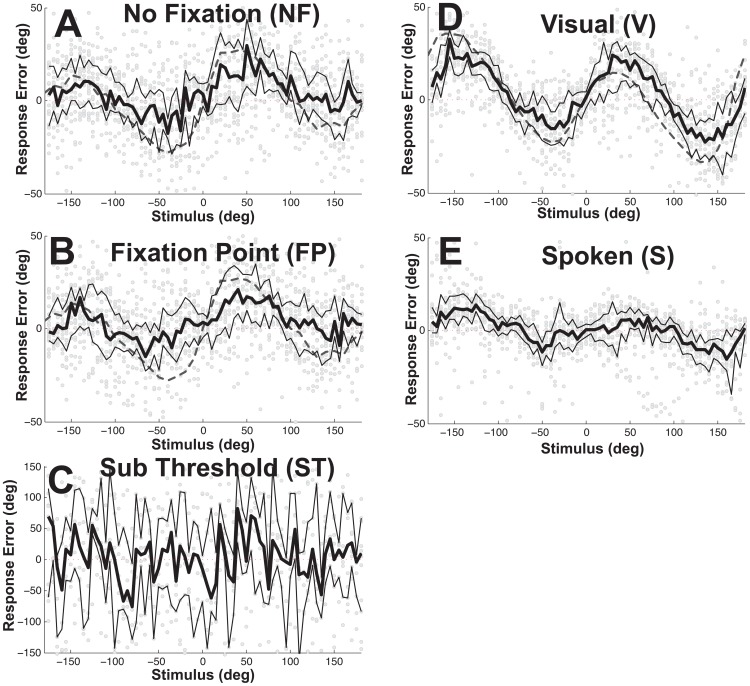
Error in perceived heading as a function of stimulus heading. Ideal performance would be represented by a horizontal line at zero. Each panel represents a stimulus type. Combined data is shown for all subjects. Individual responses are shown as gray circles. The median of the individual responses is shown as a dark solid line. Thin lines represent the 25^th^ and 75^th^ percentiles. The angle zero represents straight ahead. Panel A: 16 cm displacement in darkness, no fixation point was present. Dashed line represents the theoretical performance previously predicted by a population vector decoder (PVD) model [8]. 2% of data points are outside the range shown. Panel B: 16 cm displacement with a fixation point visible at eye level, 2% of data points are outside the range shown. Panel C: 1 cm displacement in darkness, no fixation point was visible. Note that range of errors shown are ±150 degrees, 12% of responses were outside this range. Panel D: Visual motion through a star field with binocular disparity, the visual motion stimulated a 16 cm displacement but no platform motion occurred. 5% of data points are outside the range shown. Panel E: Spoken commands were given in darkness and the subject oriented the dial to the requested heading. 4% of data points are outside the plotted range.

**Figure 2:**
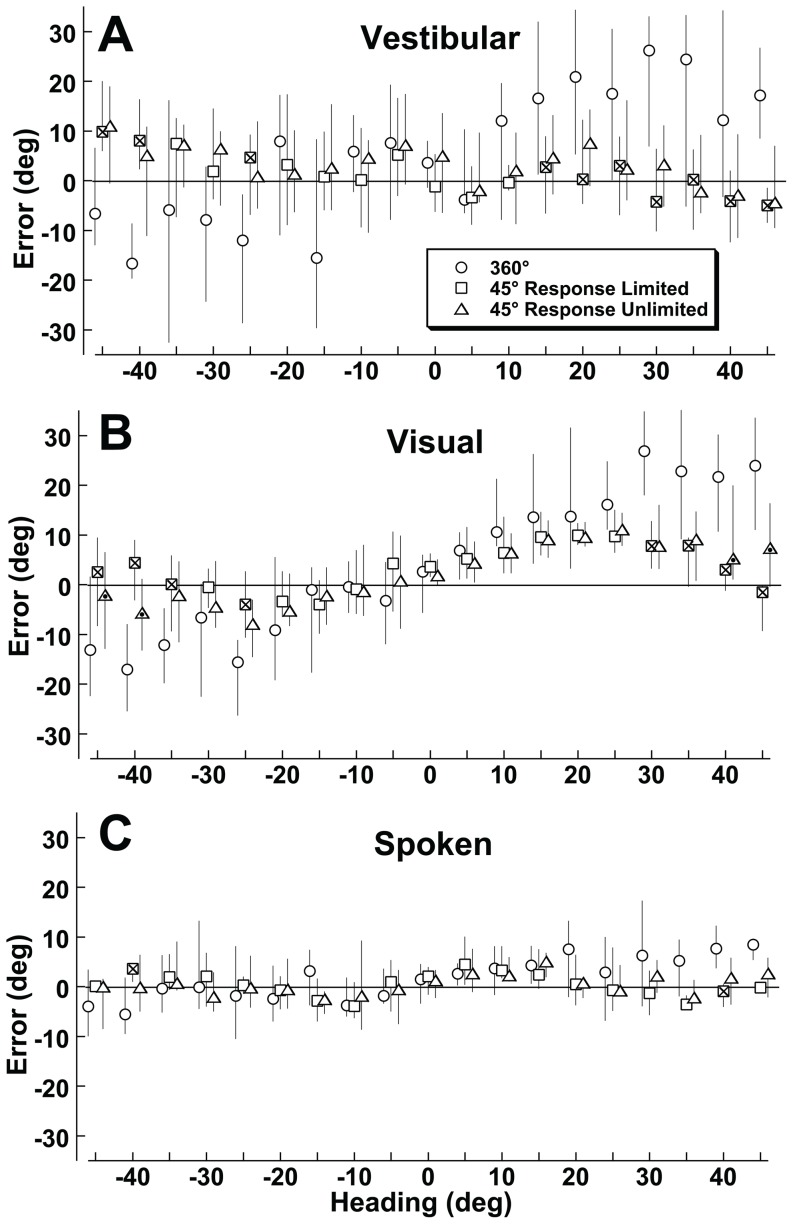
Effect of limiting headings to ±45°. In a subset of 6 subjects (#1,2,3, 7, 8, and 9) the task was repeated with the possible headings limited to ±45°. This task was initially done with the responses mechanically limited to ±45° (squares) and subsequently in the same session with the full range of responses available (triangles). Each data point represents the median response with error bars representing 25^th^ to 75^th^ percentiles. The responses with a full range of possible headings and responses are shown as circles, these are the same responses plotted in Fig. 1 but for the subset of subjects who also completed the limited heading task. Squares marked with ‘X’ indicate the perceived headings during the 45° response limited condition were significantly different than the perceived headings when the full range of headings was delivered. Triangles marked with dots indicate a significant difference (p<0.01) between conditions where the responses were limited to ±45° and the condition where only the headings were limited to ±45°, this was uncommon and limited to the visual stimulus for headings of ±40° and ±45°.

**Figure 3:**
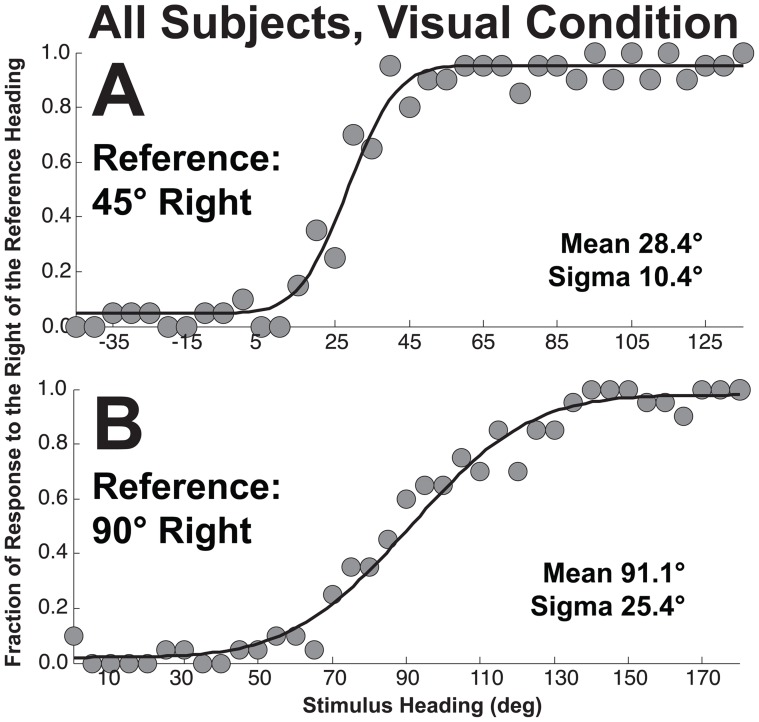
The psychometric method of determining perceived heading for two sample headings. The plot contains data from all 10 subjects each of whom completed 2 blocks of trials with the visual stimulus. Thus, each data point represents 20 stimulus presentations. For each plot a reference heading was chosen and stimulus headings at ±90° relative to the reference heading were considered. The ordinate represents the fraction of responses to the right of the reference heading. With ideal performance, all the headings to the left of the reference stimulus heading would be 0 and all the headings to the right of the reference would be one. The solid line represents the cumulative distribution function which best approximates the data. Panel A: Reference heading is 45° right. The cumulative distribution function predicts that the point of subjective equality (PSE) at which the stimulus is equally likely to be perceived left or right of 45° would be 28.4°. Panel B: The reference heading is 90° right. In this example a perceived heading of 90° is most likely with a stimulus of 91.1°. Although the accuracy of the perceived heading is better, the precision (sigma) is worse when compared with 45° in panel A.

The dial setting was compared with the actual heading for each trial to calculate an error (Figs. 1 and 2). This direct method allowed the error to be calculated across subjects. Due to the large number of headings (72) and the limited number of repetitions of each heading simply taking the average at each heading was susceptible to noise when applied to the data of an individual subject. The averaging method also does not provide a reliable measure of precision (reproducibility) of responses since there were small numbers of trials at each heading. These issues were addressed using a psychometric technique: Each of the 72 possible stimulus headings was used as a reference heading. The responses to all the headings within ±90° of this heading were examined to determine if the response heading were right or left of the reference heading. A cumulative distribution function could then be fit to these responses (Fig. 3) using the technique previously described [38]. Each fit was reiterated 100 times using resampled responses to permit determination of confidence intervals [38]. A lapse rate of 0 to 0.05 was fit to the responses. Using this method, for each reference heading the mean of the psychometric function or point of subjective equality (PSE) represented the heading at which subjects were equally likely to perceive a heading left or right of the reference heading. The width of the psychometric function (sigma) represented a measure of the precision or reproducibility of the responses.

Statistical significance of other types of responses was determined using ANOVA in Prism (GraphPad, La Jolla, CA) with the threshold for significance set at p<0.01. Correlations between continuous variables were analyzed using a two-tailed Spearman’s rank order correlation coefficient or when more than two continuous variables were compared partial correlation coefficients.

## Results

The data combined across subjects are reported first so the general trends can be described before exploring individual variation. The perceived heading had a direction dependent bias for all test conditions except ST (Fig. 1). There was a tendency to overestimate the lateral aspect of movement: Headings to the right of the fore-aft axis were estimated further to the right and those to the left were estimated further to the left. The effect was most pronounced with conditions NF, FP, and V in which 16 cm displacement was used (Fig. 1A,B, and D). Thus, for headings between straight ahead (0°) and pure rightward movement (90°) the heading was estimated further to the right than the actual heading (more positive). For headings with a forward and leftward component (0 to −90°) the heading was estimated as further to the left than actual (more negative). For movements with a significant backward component this trend was similar such that headings were perceived as more lateral than they actually were. The spoken (S) stimulus also demonstrated a direction specific bias in perceived heading although it was less than seen for the NF, FP, and V conditions (Fig. 1E). The trend in perceived heading errors was similar to that predicted from a PVD model of MSTd in monkeys [8] with the model predictions shown as a dashed line in Fig. 1A and B for vestibular motion and 1D for visual motion. The ML model predicts no bias, so this model would represented as a flat line at zero.

The sub-threshold (ST) condition (Fig. 1C) produced responses that were essentially all noise. This data was collected to investigate the possibility that subjects might guess certain headings (i.e. the cardinal directions) more frequently but this was not the case. Although further analysis was performed on this condition because the data was essentially all noise, no further analysis of ST is presented.

Care was taken to see if presence of a visible fixation point (FP) during platform motion had any effect when compared with motion in darkness with no fixation (NF). These two conditions produced qualitatively similar results when examined across subjects (Fig. 1A and 1B). In the combined data the standard deviation of the direction specific bias was not different between the two conditions (paired T-test, p = 0.12). There was also a tight correlation between direction specific heading errors in two conditions (R = 0.93, slope 0.95, p<<0.001). Because the two conditions produced virtually identical results, further analysis of the FP and NF conditions will be reported for the combined data set referred to as the ‘vestibular’ condition.

Analysis was performed to see if heading estimation changed with subsequent exposure to the task. This was done by combining the data across all 10 subjects for the first trial block and comparing it with the data for the final trial block. For the visual headings the bias on the first block of trials was highly correlated with the bias found on the second (R = 0.98, slope 0.91) and a paired-test demonstrated no different between the biases (p = 0.35) or sigma (p = 0.13). With the vestibular conditions the FP and NF trials were combined so that the first trial block of vestibular heading perception could be compared with the forth trial block. Here the absolute amount of bias was slightly greater on the first attempt at 7.2° vs. the forth attempt at 5.4° but this difference was not significant (paired T-test, p = 0.07). The precision (sigma) was similar between the first and forth attempt at 17.4° vs 17.6° (p = 0.84). For the S condition the bias in the first block was tightly correlated with the bias on the second (R = 0.93, slope 0.98) with no significant difference in bias between the two blocks (T-test, p = 0.95) or sigma (p = 0.32). Thus previous experience to the task did not have an appreciable influence on subsequent performance.

Heading perception was also measured in a separate block of trials over the limited range of ±45° in the NF, V, and S conditions (Fig. 2). Limiting the range of stimuli had the effect of decreasing direction specific biases regardless of the range of responses permitted. The decrease in bias was most pronounced and significant at the more eccentric headings where the bias was larger (Fig. 2). When compared with heading perception when a full range of headings was used the direction specific bias was significantly smaller at eccentric headings for the NF and V conditions.

**Figure 4:**
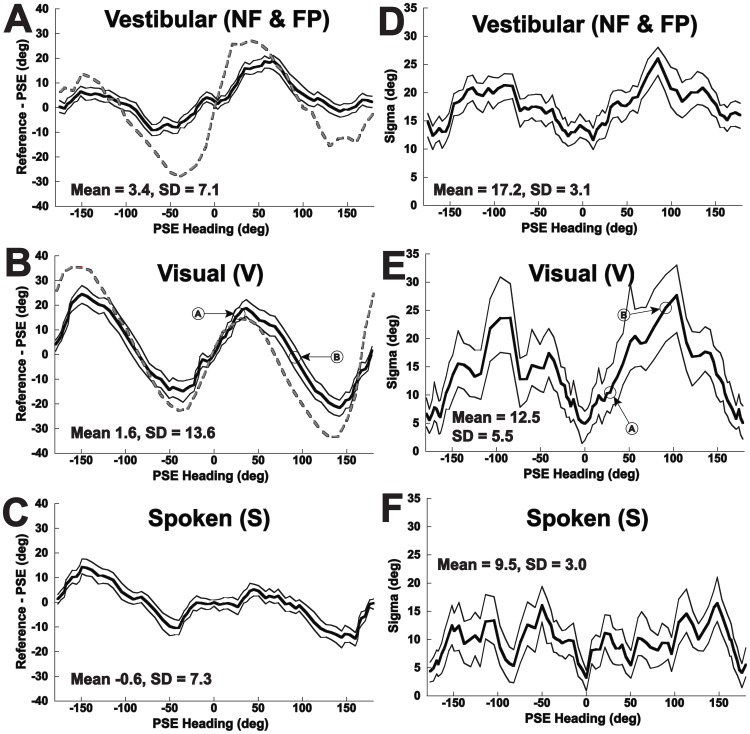
Direction specific bias (panels A–C) and precision (sigma, panels D–F) for perceived heading across subjects. The results are qualitatively similar to those in Fig. 1, but calculated using the method in Fig. 3. The previously predicted performance based on a PVD model [8] is shown as a red dashed line for the vestibular and visual conditions. The thick line represents the mean value, and the two thin lines represent the 95% CI based on 100 fits with resampled data in each iteration. Panel A: Vestibular motion. Panel B: Visual motion (optic flow). The data points shown calculated in Fig. 3A&B are marked with arrows. Panel C: The subject orients a dial based on a spoken heading. Panel D: Precision for the vestibular condition. Panel E: Precision for the visual stimulus, points calculated in Fig. 3A&B are marked. Panel F: Precision for the spoken condition.

A psychometric technique was applied to determine the precision of responses allowing them to be more directly compared with prior experiments on heading discrimination. This technique also decreased the noise in the heading estimates by using a 180° range of headings to determine the bias for each heading direction, but could only be used in conditions where the full range of headings was tested. Applying the psychometric technique described in methods to the combined responses demonstrated that classification of responses based on relative directions was closely approximated by a cumulative distribution function (Fig. 3). Results of these psychometric fits (Fig. 4) demonstrated the mean error between perceived and stimulus headings was similar to simply averaging the response (Fig. 1) but with less noise and also allowed determination of sigma as a measure of precision. The amount of direction specific error in heading perception could be quantified by taking the difference between the reference and the PSE across the range of headings (Fig. 4A–C).

The precision of perceived headings was determined using the width (sigma) of the psychometric function that best fit the responses (Fig. 3). This precision was best for the spoken condition, followed by the visual and vestibular conditions (Fig. 4D–F). For each condition the precision was best for headings close to straight ahead (0°) and straight backwards (180°). For the vestibular and visual conditions the precision was considerably worse for more lateral headings (i.e. near ±90°) consistent with previous results using a discrimination task [8].

**Figure 5:**
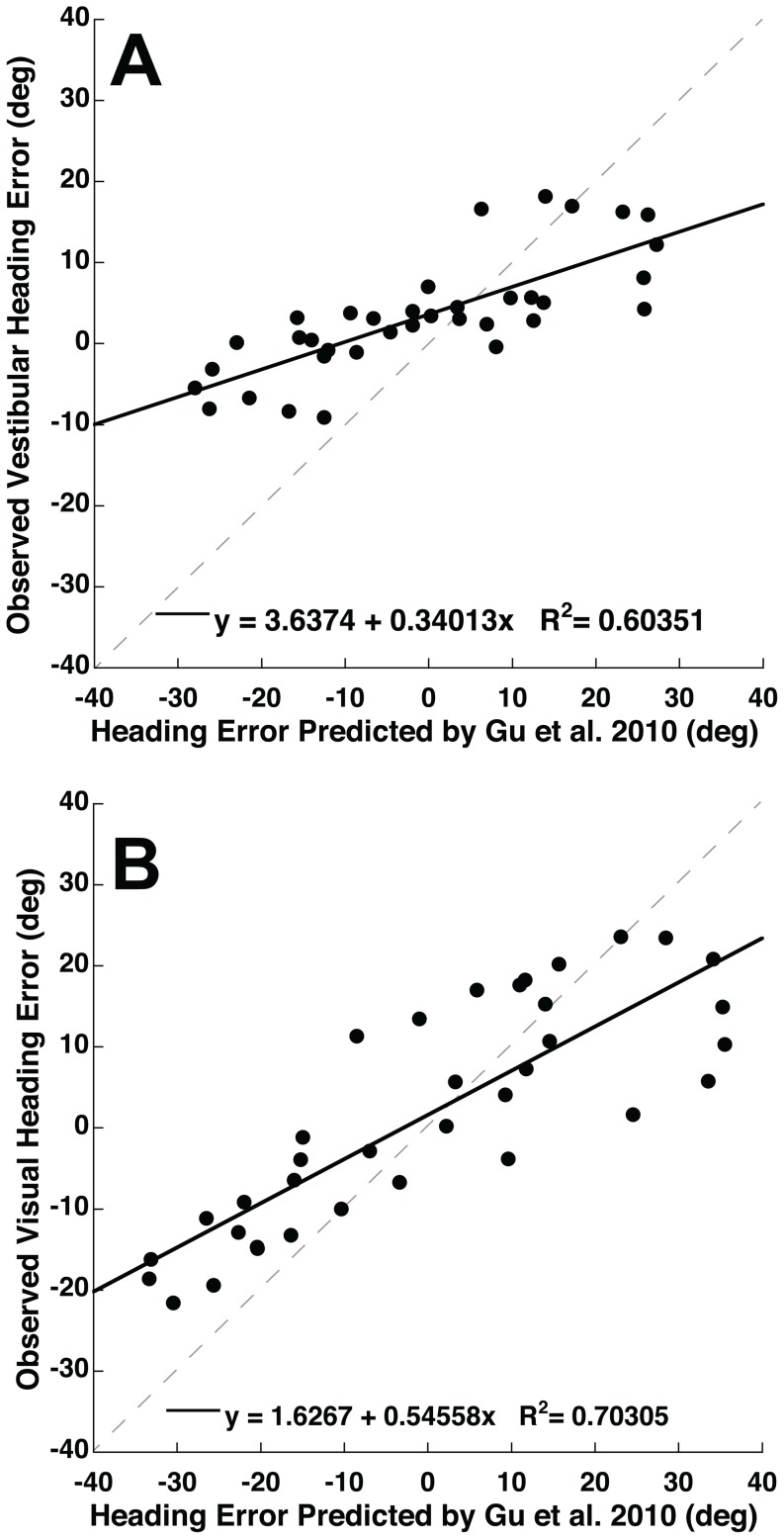
Correlations between the PVD model predictions [**29**] and experimental observations of heading perception. The perceived headings were calculated using the method in Fig. 4. The best-fit linear regression is shown as a solid line on each panel. The gray dashed line represents unity. When the heading errors are compared there was a correlation for the vestibular (panel A) and visual (panel B) conditions.

To further evaluate the observed heading errors relative to a model, a linear regression was performed between the observed headings and the model predictions. There was a strong positive correlation between the model prediction for heading and the perceived heading for both vestibular and visual conditions (R^2^>0.98 with slope near unity for both). When just the heading error was considered (Fig. 5A&B) the correlation was less strong with R^2^ = 0.60 (Fig. 5A, p = 0.0001) for vestibular headings and R^2^ = 0.70 (Fig. 5B, p<0.0001) for the visual condition. The slope of the linear regression was 0.34 for vestibular and 0.55 for vision indicating the observed biases were generally smaller than those predicted by the model.

**Figure 6:**
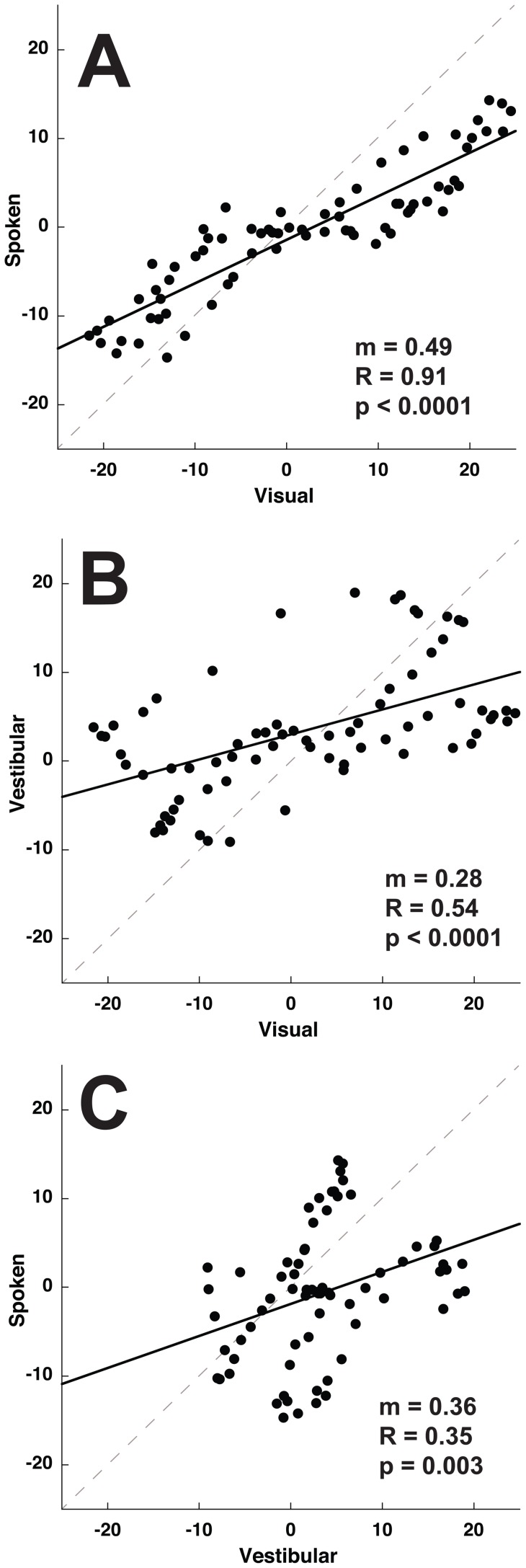
Correlations in direction specific bias between subjects for 3 stimulus conditions. Data was generated from responses combined across subjects. There is one data point for each of the 72 stimulus headings. The same error could occur at multiple headings so any given error in one sensory modality could correspond to multiple errors in another. Slope (m), Correlation coefficient (R), and p-value are given for each condition. A solid line represents the best-fit linear regression. The gray dashed line represents unity. Panel A: Visual-spoken. Panel B: Visual-vestibular. Panel C: Vestibular-spoken.

Correlations between the direction specific biases were found in the visual, vestibular, and spoken conditions. These correlations using the data pooled across all ten subjects are shown in Fig. 6. The strongest correlation was between the visual and spoken condition (Fig. 6A, R = 0.91, p<<0.001) with the direction specific biases for the spoken condition being half that of visual headings. This difference in magnitude was evident from the slope of the linear regression (m = 0.49) and the standard deviations of the bias (13.6 for visual vs. 7.1 for vestibular). Thus the direction specific bias curves for the visual and spoken conditions had a similar shape although the bias was smaller in the spoken condition. Although correlations between the visual-vestibular and vestibular-spoken conditions remained significant (Fig. 6B and 6C) they were weaker. This poor correlation was due to the vestibular condition tending to have a direction specific bias that was large for headings with a forward component but much smaller for headings with a backward component.

**Figure 7:**
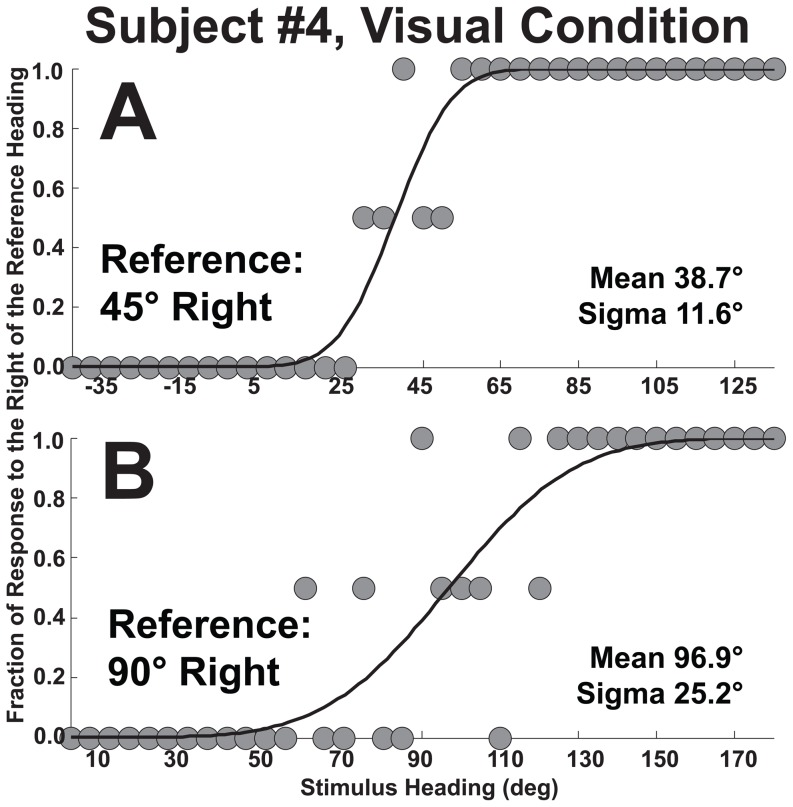
The psychometric method of determining perceived heading. This example is for the visual stimulus in subject #4. Each point represents 2 stimulus presentations. The figure is analogous to Fig. 3 except data from a single subject is shown. Panel A: The reference heading is 45° right. Panel B: The reference heading is 90° right.

**Figure 8:**
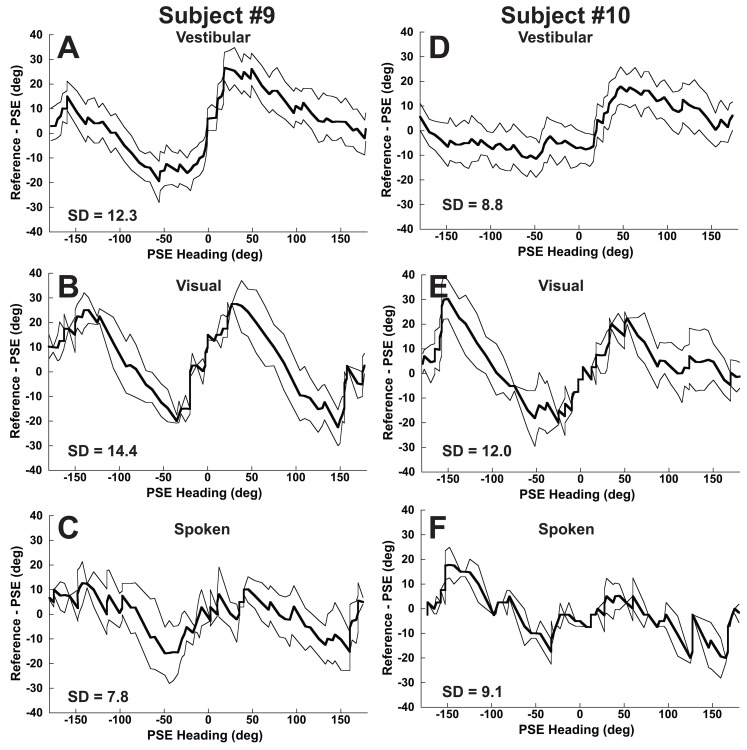
Direction specific bias for two subjects (#9 and #10) for three stimulus conditions. These plots are analogous to Fig. 4A–C, but are for specific subjects rather than the whole population. The thick line represents the mean value, and the two thin lines represent the 95% CI based on 100 fits with resampling of the data with each iteration. Panels A and D: Vestibular motion. Panels B and E: Visual motion (optic flow). Panels C and F: The subject orients a dial based on a spoken heading.

Further analysis will focus on individual subjects to determine to what degree these trends were observed. The psychometric fitting technique was robust enough to apply to individual subjects (Fig. 7) permitting individual direction specific bias curves to be determined (Fig. 8). The trend of overestimating the lateral heading component was often similar to that seen in the combined data with biases in the direction of heading but with significant variation in the magnitude of the bias between subjects (i.e. Fig. 8A vs. 8D). The visual and spoken heading curves often had a similar shape for each subject. For instance, in subject #10 there was a trend towards a larger bias in heading perception for visual headings with a backward and left component (−90 to −180°, Fig. 8E). In such cases there was often a hint of a similar direction specific bias in the spoken condition (Fig. 8F), with fewer consistent similarities between the visual and vestibular conditions (Fig. 8A and B vs. 8D and E).

**Figure 9:**
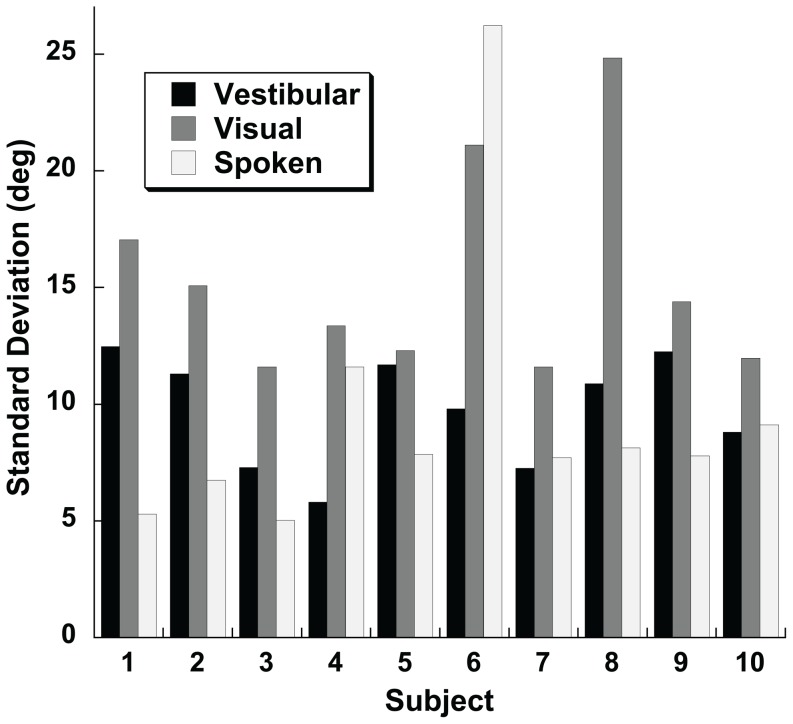
Standard deviation of direction specific biases by subject for the visual, vestibular, and spoken heading estimation conditions.

**Figure 10:**
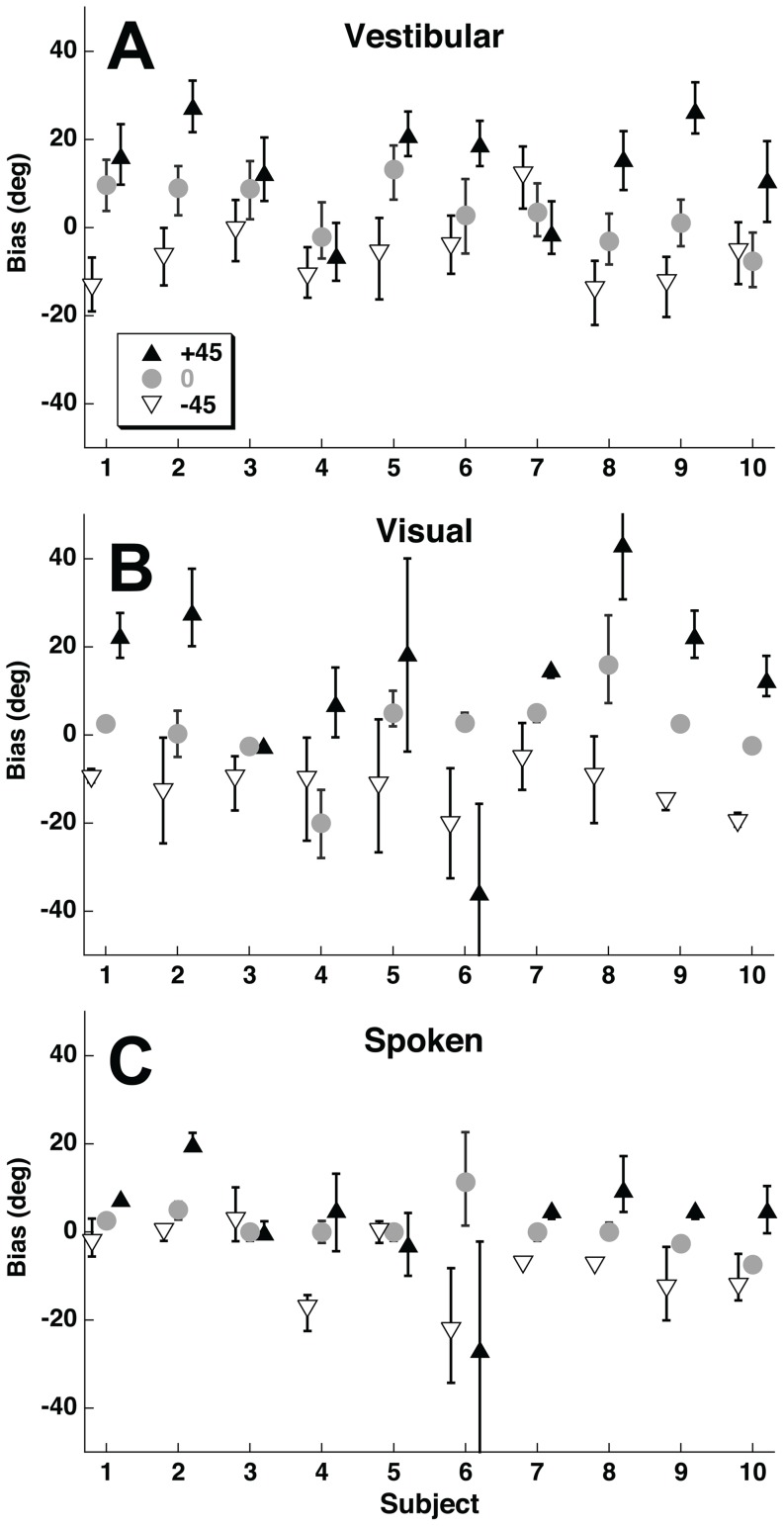
Biases for each subject at the reference headings of −45° (upward pointing filled triangle), 0° (gray circle), and 45° (downward pointing open triangle). These biases were calculated using the psychometric method shown in Fig. 7. Error bars represent 95% confidence intervals. Panel A: Vestibular headings. Panel B: Visual headings. Panel C: Spoken headings.

The amount of variation in the direction specific bias was quantified by taking the standard deviation (SD) of the bias across stimulus heading. Values for example subjects are shown (Fig. 8). For every subject the direction specific bias was greater in the visual condition than the vestibular condition (Fig. 9). The visual condition also had a greater direction specific bias when compared with the spoken condition in all but one subject. The magnitude of the variation in direction specific bias in one condition was not correlated with that found in other stimulus conditions (Pearson correlation coefficient, p>0.1 for all), thus subjects with larger biases in the visual condition did not tend to have larger biases in the spoken or vestibular conditions and vice versa. Individual variation in biases amoung subjects was examined by looking at biases for each subject at ±45°, and 0° (Fig. 10). For the vestibular and visual conditions 8/10 subjects had positive biases with a 45° stimulus and negative biases at −45°. With the spoken condition the biases were more variable between subjects but were usually smaller.

**Figure 11:**
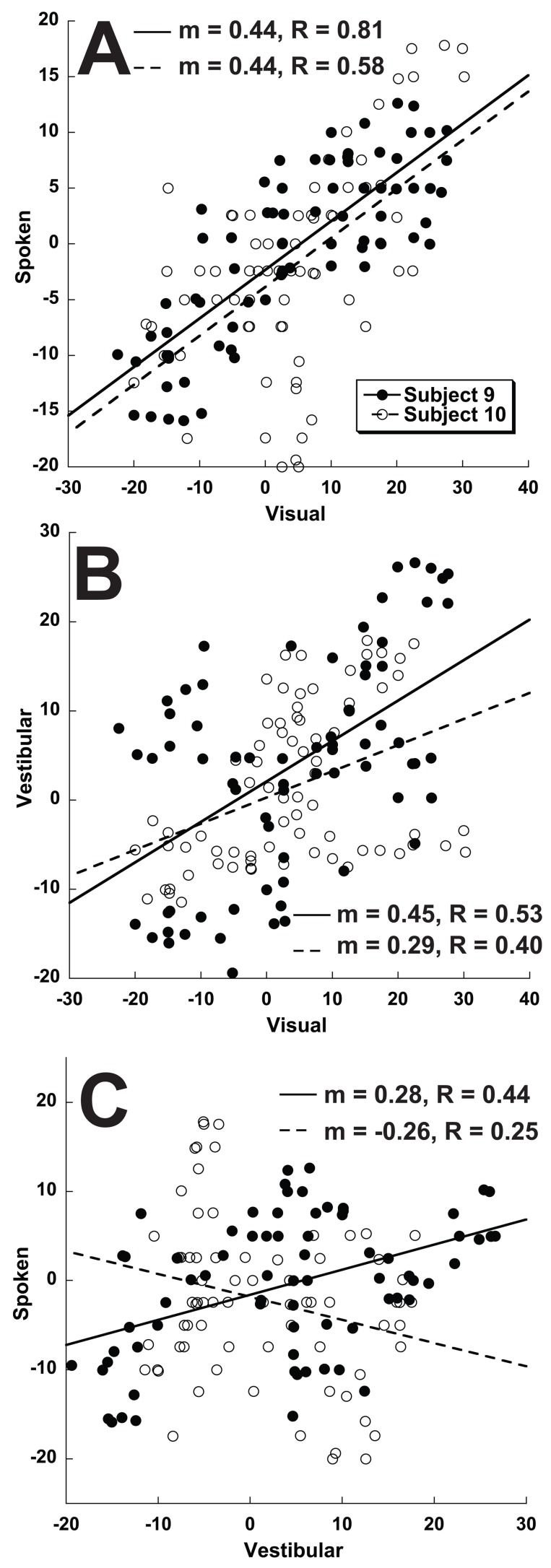
Correlations for heading dependent bias paired across heading estimation tasks from different sensory modalities. Data shown are for subject #9 (filled circles) and #10 (open circles). These are the same subjects and data shown in Figure 6, but re-plotted to demonstrate correlation. For the number of data points p<0.001 for R≥0.38 thus all the correlations shown are highly significant except for subject #10 in panel C (p = 0.03). Panel A: Visual-spoken. Panel B: Visual-vestibular. Panel C: Vestibular-spoken.

**Figure 12:**
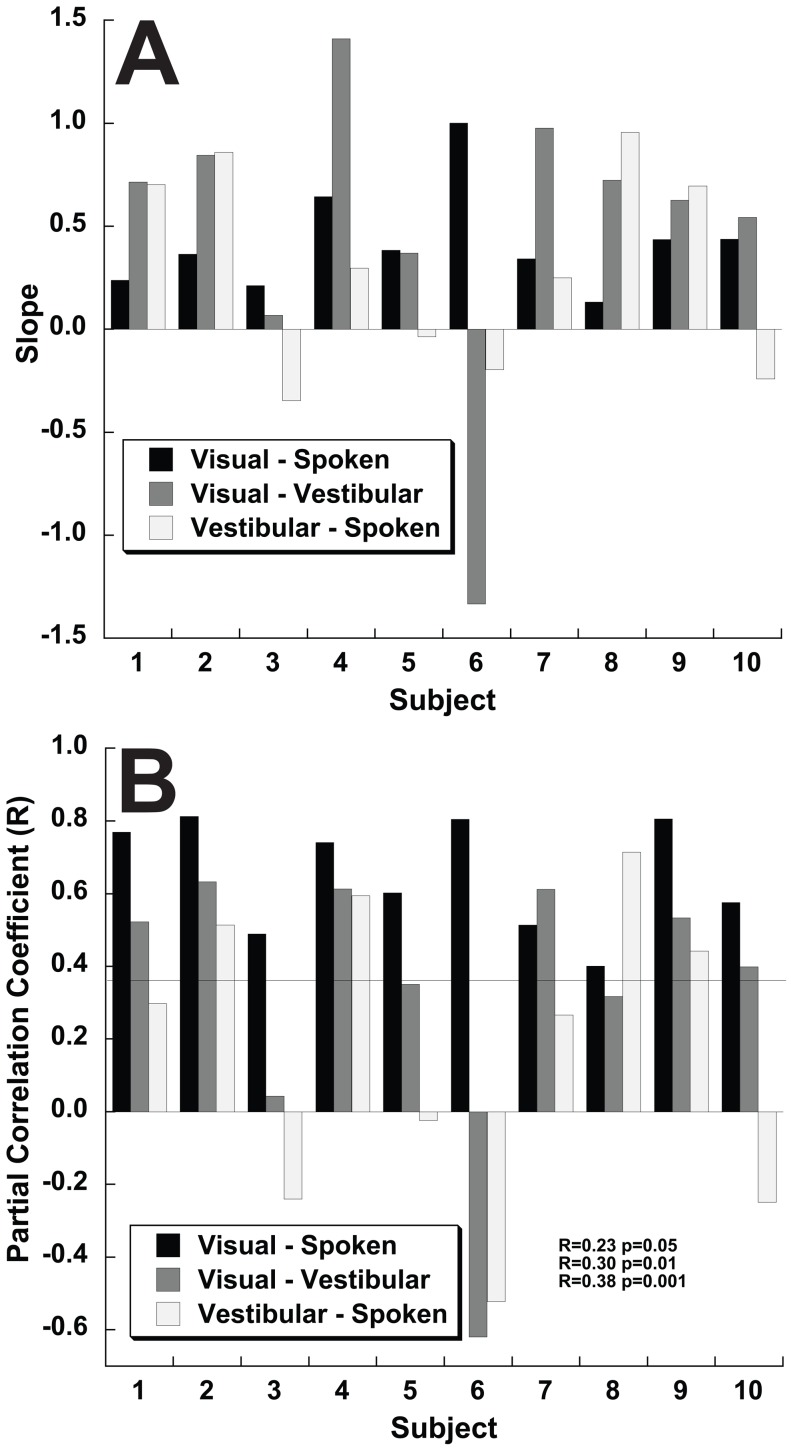
Correlations in heading bias between the 3 test conditions by subject. Panel A: Slope of the correlation. Panel B: Partial correlation coefficient (R). For R>0.38 (line in panel B) the correlation is highly significant (p<0.001), and R>0.30 remains significant (p<0.01).

Even though the magnitude of the biases was not correlated, potential correlations in shape of the bias curves was explored by determining the slope and correlation coefficients between each of the 3 test conditions (Fig. 11). Similar to that seen in the combined data, there was a highly significant correlation between the direction specific biases in the visual and spoken condition in every subject (Fig. 12). The slope of the correlation between visual and spoken conditions was consistent with the spoken condition having a smaller bias in almost all subjects (Fig. 12A, mean m = 0.42±0.25, range 0.21 to 1.00). The correlation coefficient was highly significant in every individual (Fig. 12B, mean R = 0.65, range 0.40 to 0.81), for these correlations with 72 data points, p<0.001 for |R|>0.38.

The correlations between visual-vestibular and vestibular-spoken perceived direction dependent heading biases were weaker and less consistent than those seen with visual-spoken condition. For the visual-vestibular condition 9/10 subjects had a positive correlation but the slope was more variable (Fig. 12A, m = 0.49±0.73, range −1.33 to 1.41) and the correlation coefficient lower in all but one subject when compared with visual-spoken (Fig. 12B, mean R = 0.34±0.28). Subject #6 was an outlier with a large inverse correlation between visual and vestibular bias curves. The atypical results in this subject may have been related to difficulty with the task as demonstrated by large variability in his responses. The vestibular-spoken conditions had a positive correlation in only 7/10 subjects (m = 0.29±0.49, range −0.34 to 0.95).

There was no correlation between subject age and either accuracy or precision for any of the stimuli types (Pearson correlation, p>0.05 for each). Also no effects of gender or handedness were seen but the study only included 3 females and 1 left handed individual.

## Discussion

The current data establish that humans overestimate the lateral component of heading with both visual and vestibular stimuli, and to a lesser extent spoken headings. These findings are unexpected given previous reports of visual heading estimation. Several prior studies have demonstrated that the visual heading is underestimated [10], [13], [14], or underestimated in most subjects [11]. The reason these prior studies did not demonstrate the large headings errors may be because the range of headings was limited to at most ±25°. The participants of these studies may have had prior knowledge that the potential headings were limited to this range or determined this after early stimulus presentations and may have influenced their responses accordingly. When a cursor is used to measure the heading perception the range of responses is also limited by the size of the display screen [10], [11], [13].

When the headings were limited to ±45° in the current paper (Fig. 2) the biases were found to be much diminished in line with previous papers that also tested a limited range [10], [11], [13], [15]. For visual and vestibular headings at the extreme of the range a small underestimate was observed, consistent with the small overestimate in other studies [10]. This might be explained by subjects ‘leaving room’ for more extreme stimuli by not choosing the maximal excursion. This strongly implies that limits on the range of headings tested can cause the direction specific biases to be smaller and in some situations even reverse. Limiting the stimuli had similar results with and without limits on the responses (Fig. 2).

One previous study reported large overestimates in perceived headings with a visual stimulus. A heading of 20° (the largest used in that study) was over estimated by about 25° with headings closer to straight ahead underestimated by a smaller amount [12]. It is unclear why Telford and Howard were able to see large heading biases within a limited range of headings when others did not. These authors suggested their finding could be due to their stimulus which included vertical bars rather than typical points. Although not mentioned by these authors, it is also possible that the difference may be due to the method used to collect responses did not include limitations on the perceived direction. The large biases were nearly eliminated when the subject was able to move their head freely and in vestibular heading conditions in the same study. The order the stimuli were tested was not specified in that study but it is possible that subjects ‘discovered’ the range of possible headings was smaller than the initial estimate during the course of the experiment, which could have influenced subsequent perception. The current study demonstrates that the lateral aspect of visual heading is often greatly over estimated. The reason for this finding is likely because neither the range of possible stimuli nor the range of responses was limited by the experimental design, thus the subject’s prior probability distribution was less likely to be limited.

The current study demonstrates that vestibular heading was also overestimated relative to the fore-aft axis. To our knowledge only one previously published study has found large overestimates in heading, which was discussed above with regard to visual heading [12]. However other studies have demonstrate overestimates of about 5° [15] and overestimates in 1 of 8 subjects [11]. Although Telford and Howard found findings similar to the present paper for a visual heading task, they found an opposite result for their vestibular heading task: a slight tendency to underestimate heading angles in darkness. There are several potential reasons for these differences: First, as mentioned previously, the subjects likely had prior knowledge that the range of vestibular headings would be limited to ±20° due to the design of the experiment in which subjects were given eight practice trials with feedback prior to collecting data and limited their responses accordingly. Second, the translation occurred along a fixed track with the subject rotated prior to translation, so there may have also been other cues to their orientation. Third, the stimulus used also included a much longer displacement (600 cm) and longer duration (11 s) than the stimuli used here so it is possible that heading estimation depends on stimulus duration or displacement. Fourth, their stimulus did not deliver pressure to the head during the head free condition and these absent pressure cues may have altered the perception. Finally, the number of subjects in the Telford and Howard study was small (5 subjects), with few stimulus presentations per subject (18) so there was not as much power to eliminate noise from the results. It is not clear why this earlier study found vestibular headings to be slightly underestimated but it may be a combination of factors perhaps including over correction of perceptual errors after the practice trials with feedback.

In this study, the presence of a visual fixation point had no significant effect on heading perception. Although there is a rich literature demonstrating a visual fixation point decreases the threshold of rotation perception [39]–[44] a fixation point does not influence translation perception [41]. This study is consistent with the report of Benson and Brown, but also demonstrates that a visual fixation point is not needed as a reference point for heading determination.

In the visual heading experiments a fixation point was used for all trials. This was because many of the prior experiments on visual heading also used a fixation point [7], [8], [10]. If a fixation point is not used the initial eye position would not be controlled and could influence heading perception [13]. However, in studies where no fixation point was used, it was thought that the heading perception was similar to previous studies that did use a fixation point [9].

Visual heading discrimination is excellent when differentiating headings near straight ahead [5], [25], [45] but becomes much less precise for more lateral headings [3], [8]. In the current study the fall off in visual heading sensitivity with eccentric headings was not as great as previously described [3] which may be due to the larger horizontal field of view (FOV) in the current experiment (98°) which provided more optic flow information [16]. The precision of heading estimation in the current study was only slightly worse than that previously described in a forced choice discrimination technique [8] using a similar display size (90°). This difference may be due to variability in orienting the dial in the current experiments, which would not be an issue in a forced choice discrimination task.

The accuracy of heading perception is likely related to the size of the FOV with heading being theoretically nearly impossible to determine with the FOV <2–3° and reaching a theoretical maximum about 100° [16]. Although the FOV used in the current study at 98° was larger than that in some previous work on heading estimation – i.e. 45° [10] or 60° [12], the FOV did not include the full visual field and covered only 27% of the range of headings (360°). Thus for the visual headings at 50° and further lateral the FOE was not visible. It has previously been suggested that triangulation error may cause overestimation of headings from optic flow when the FOE is outside the FOV based on postural data [46] as well as heading perception [11]. The Li et al. study on heading determination found overestimation in only 1 of 8 human subjects and that subject had advanced retinitis pigmentosa and was consciously aware of the strategy [11] suggesting that this strategy not generally applicable. A study which included large headings of up to 90°, many of which had a FOE outside the 53°FOV also found overestimates but, as with the current data, this was true even for headings with the FOE within the FOV [15]. Triangulation error predicts that the FOE will be estimated as too lateral only when the FOE is not visible because when it is visible it may be directly identified or estimated using velocity vectors calculated from fiducials on both sides of the FOE. In the current data the bias is seen well before the limits of the FOV (Fig. 2B and 4B) so the bias cannot be explained with errors in triangulation. Furthermore, in the current data the overestimation bias reaches a maximum at about 45° (Fig. 4B) and the bias decreases with further lateral headings out to about 100°. A similar effect has also previously been described for headings in the range of 0 to 90° [15]. These observations are not consistent with this bias being due to triangulation error as that theory predicts the maximum bias near 90°. Thus the biases seen in visual heading estimation are not likely to be related to the size of the FOV or triangulation of the FOE when it is outside the FOV.

How the heading is reported is a potentially important issue in interpreting heading estimates. In prior studies the perceived heading has been reported either with a cursor on a screen which allows the subject to make a direct mapping between the perceived heading and the visual display [10], [11], [13] or by orienting an object in space which is potentially more dependent on haptic influences [12], [15]. In the current study, the second method was used because the full range of headings cannot be represented within the visual field, and this technique was felt to be more appropriate for reporting non-visual heading perception. Using either of these techniques the measured headings are also potentially influenced by haptic and motor systems which could also influence the bias estimate. One way to eliminate this influence would be to study heading discrimination - i.e. using a forced choice task to report the direction of a test stimulus relative to a reference stimulus or position (i.e. straight ahead). Although discrimination methods have been used extensively [2], [7], [8], [34], they do not permit measurement of bias. In the current study several control experiments were conducted to measure the potential bias introduced by these haptic and motor influences. First a subthreshold (ST) vestibular stimulus was used to see if subjects had a tendency to choose some headings over others (Fig. 1C) which was not the case. Second, the perceived direction of spoken headings were measured to provide a metric for spatial cognition, motor, and haptic biases independent of sensory manipulation. If the pattern of biases seen with visual and vestibular heading estimation (Fig. 4A&B) were similar to those measured using a spoken stimulus (Fig. 4C) it would imply that the observed biases are primarily due to motor or haptic biases but this was not the case. Although there was some bias in the spoken condition it was minimal for angles near straight ahead, much less than was seen for visual and vestibular stimuli at other angles. Third, when the spoken, vestibular, and visual conditions were repeated using a limited range of ±45° (Fig. 2) the limited range had a much greater influence on the visual and vestibular biases than the biases in perception of spoken headings. Thus it seems most likely that the major influence on the biases was related to heading estimation from sensory stimuli rather than motor or haptic issues.

An interesting result of this study is that for the spoken heading condition there was very little bias for headings near straight ahead. Over ±20° the average bias was <1° at every heading even when a full range of headings was presented (Fig. 4C). However, there was significant direction specific bias for both the visual and vestibular conditions within this range – at 20° the mean visual bias was 7.3° and the mean vestibular bias was 4.3°. Thus, when the perceived headings with the spoken heading are correlated with visual (Fig. 6A) or vestibular (Fig. 6C) there appears to be a non-linear correlation in biases near 0°. This may be evidence for a prior expectation of a straight ahead movement or it may be due to better tuning towards straight ahead since this is most of our day-to-day experience.

There is evidence that area MSTd is key to both visual and vestibular heading estimation [25], [27], [31], [47]. Within MSTd not all headings are represented equally [47], [48], and recent modeling using a population vector decoder [30], [32], [49] has demonstrated that this can explain why both visual and vestibular heading discrimination becomes worse with more eccentric trajectories [8]. This model also predicted that both visual and vestibular heading estimates would have large biases due to overestimation of the lateral component of the heading vector. With regard to these predictions, Gu et al. concluded that, “it is unlikely that humans or monkeys exhibit behavioral biases in heading estimation as large as those predicted by the population vector decoder, but at present there is no data to verify or contradict this assertion.” [8] However, the current paper tested their predicted biases and demonstrated a trend similar to that predicted by their PVD model (Fig. 1A, B, D, Fig. 4A, B, and Fig. 5) for both visual and vestibular heading estimation.

There were some interesting differences in the predictions of the PVD model [29] and the observed headings. The predicted trend in vestibular (Fig. 4A) heading estimation, although qualitatively similar, was not as large as those observed as can be seen by the slope of 0.34 (Fig. 5A). It was also of interest that the model predicted a larger bias for headings with a forward component (headings of −90 to 90°, Fig. 4A) than those with a backward component but this fore-aft dependent difference bias was not evident in the data. For visual headings the model predicts an opposite trend in biases such that direction specific biases would be larger for backward motion (Fig. 4B), which was also not observed here. The reasons for this may be due to species differences or individual variation as the variation between subjects was considerable even in the current human data and the Gu et al. model was based on a sampling of individual neurons in two monkeys. It is also possible that changes outside of MSTd influenced perception that were not predicted by the model. However the similar trends between the PVD model predictions and the observed visual and vestibular heading errors suggest that these heading errors may be explained by the mechanism suggested by the PVD model.

The relevance of the PVD model presumably does not depend on the range of headings tested, yet the direction specific biases seem to be minimized in studies where a limited range of headings were tested [10], [11], [13], [15]. In the current study some overestimates in heading estimation were seen in the middle of the range tested (see 20–25° in Fig. 2) and similar behavior was also seen by others when the raw responses were considered [10]. These overestimates away from the limits of the range of test stimuli suggest that the PVD model is still relevant. It is likely that these large biases are masked by the subject’s estimate of the range of stimuli when they have knowledge or gain knowledge during the experiment that the range of headings is limited.

Although our data is consistent with these biases occurring as a result of the known physiology in MSTd, there are also other possibilities. The population decoder model predicts that these biases are caused by over-representation of units with sensitivity to lateral headings relative to those with sensitivity to fore-aft headings, but this over-representation could also occur in other areas. Lateral headings are also over represented in the otolith end organs. In the monkey ¾ of otolith afferents respond to ipsilateral tilt [50] and the utricle orientation in human anatomy also suggests better sensitivity to lateral motion [51], [52].

The possibility that observed direction specific biases may have been caused by a skewed internal representation of space was considered. The response to sub-threshold (ST) stimuli (Fig. 1C) did not demonstrate a preference for certain headings. This made it less likely the observed biases were related to positioning of the dial alone. The spoken heading task was conceived as an estimate of each subject’s internal spatial representation. The spoken biases leave open the possibility that the observed biases are, at least in part, due to a distortion in spatial representation. However, the direction dependent biases to spoken headings, were usually smaller than those seen for visual and vestibular stimuli (Figs. 1E and 4C). It is possible that the larger bias in heading with visual and vestibular conditions is due to the sensory bias being combined with the internal bias.

Since the spoken, visual, and vestibular tasks had qualitatively similar direction specific biases, it is possible that the larger direction dependent biases seen during visual and vestibular heading estimation relative to the spoken condition could derived from an increase in a gain factor perhaps due to a more realistic stimulus during the visual and vestibular conditions. This possibility was investigated by measuring the correlation between the shapes of these bias curves (Figs. 11 and 12). There was a strong correlation between the shape of the bias curve during spoken and visual conditions although the amplitude of the biases was smaller in the spoken condition in 9 out of 10 subjects. The similar shape of visual and spoken bias curves within each subject despite variation between subjects suggests that the visual and spoken heading estimation tasks have a commonality that is lacking between these modalities and the vestibular task which had a weaker correlation with both the spoken and visual tasks in almost every subject (Fig. 12B). One possible explanation for this finding is that during the spoken condition subjects may imagine a visual heading potentially evoking a common mechanism. Given that the known physiology in MSTd can explain the observed heading biases [8] it is less likely that there is a separate mechanism causing a smaller but otherwise similar bias in the spoken condition. The observation that the correlation in the direction specific biases appeared to be a different shape in the vestibular condition may reflect direction specificity in the vestibular end organs [50].

The current data demonstrate that human heading perception is biased causing both visual and vestibular eccentric headings to be overestimated. This may go unnoticed during ordinary behavior when heading estimation includes multiple sensory stimuli and is accompanied by immediate feedback. This bias likely occurs as a result of having better sensitivity to changes of heading relative to straight ahead. There are obvious advantages to having heading discrimination best near straight ahead since this is the heading we most commonly experience, and detection of slight deviations from it (such as when driving or running down a narrow path) is important. This could explain why MSTd as well as the otolith organs have a disproportionate number of units that are sensitive to the lateral component of motion. A PVD is a relatively straightforward and computationally efficient method for the central nervous system to interpret these heading signals, but has the disadvantage of causing biases when a disproportionate number of units are tuned to a detect lateral motion. Although a ML model would avoid such biases in visual and vestibular heading estimation [8] it would be computationally more complex, and the current observations argue it is not used. The current findings argue that for heading perception the central nervous system adopts a strategy that is more computationally efficient rather than one that would avoid bias. There are certainly possible evolutionary pressures for heading estimation to be accurate over a range of angles, for instance when aiming at prey or when choosing to follow a new course. However, during most such situations there is immediate feedback which may make the types of biases seen in the current study less relevant for natural activities, where speed may be paramount.
